# Users' guides to the medical literature: how to use an article about mortality in a humanitarian emergency

**DOI:** 10.1186/1752-1505-2-9

**Published:** 2008-09-30

**Authors:** Edward J Mills, Francesco Checchi, James J Orbinski, Michael J Schull, Frederick M Burkle, Chris Beyrer, Curtis Cooper, Colleen Hardy, Sonal Singh, Richard Garfield, Bradley A Woodruff, Gordon H Guyatt

**Affiliations:** 1 Simon Fraser University, British Columbia, Canada; 2 Department of Infectious and Tropical Diseases, London School of Hygiene and Tropical Medicine, London, UK; 3 St. Michael's Hospital, University of Toronto, Toronto, Ontario, Canada; 4 Institute for Clinical Evaluative Sciences, University of Toronto, Toronto, Ontario, Canada; 5 Harvard Humanitarian Initiative, Harvard University, Boston, USA; 6 Center for Public Health and Human Rights, Bloomberg School of Public Health, Johns Hopkins University, Baltimore, MD, USA; 7 Division of Infectious Diseases, The Ottawa Hospital, Ontario, Canada; 8 International Rescue Committee, Atlanta, GA, USA; 9 Bloomberg School of Public Health, Johns Hopkins University, Baltimore, MD, USA; 10 National Center for Disaster Preparedness, Mailman School of Public Health, Columbia University, New York, USA; 11 Nutrition Branch, Division of Nutrition and Physical Activity, Centers for Disease Control and Prevention (CDC) Atlanta, GA, USA; 12 Department of Clinical Epidemiology & Biostatistics, McMaster University, Ontario, Canada

## Abstract

The accurate interpretation of mortality surveys in humanitarian crises is useful for both public health responses and security responses. Recent examples suggest that few medical personnel and researchers can accurately interpret the validity of a mortality survey in these settings. Using an example of a mortality survey from the Democratic Republic of Congo (DRC), we demonstrate important methodological considerations that readers should keep in mind when reading a mortality survey to determine the validity of the study and the applicability of the findings to their settings.

## Public health scenario

You are a physician working for an international humanitarian medical organization as head of mission. You have recently arrived in the North Kivu province in the Eastern Democratic Republic of Congo (DRC) and are conducting a health assessment of the region to inform your medical response intervention. Media reports suggest that mortality from violence are extremely high in this area of the country, but a more accurate assessment of mortality – both directly and indirectly related to violence – will assist you in setting priorities and may mandate a call for additional medical specialists.

Political agendas may distort media reports of violence and death and the quality of the evidence on which the reports rely may be low. Further, media reports are likely to omit deaths from malnutrition and infection, often the most common causes of mortality in protracted violent settings [1,2]. The need for higher quality evidence prompts you to formulate a question of public health relevance: "In the protracted conflict setting of the Democratic Republic of Congo, to what extent is mortality elevated in conflict zones compared to other countries in the region, and what is the nature of any increase in mortality?"

### The search

When searching for recent reports about mortality in the DRC both large studies broadly representing the national population and studies of the North Kivu community in which the NGO intends to implement programmes would be useful. You will follow the recommendations of the Standardized Monitoring and Assessment of Relief and Transition (SMART) initiative and seek studies of high quality [3]. You will ask a support team in the capital, Kinshasa – your own electronic access is painfully slow – to seek retrospective surveys with coverage that represent the population and the time period of interest.

Because many NGO reports will be unpublished [4], your team will contact local offices of UN agencies, as well as major data collecting NGOs such as Médecins Sans Frontières, Action Contre la Faim, and the International Rescue Committee. You also request a search of peer-reviewed and non-peer-reviewed literature using PubMed, Evidence-AID, and common electronic medical databases. In order to identify non-peer-reviewed articles, your colleague searches Relief-Web (a media and NGO repository maintained by the Office for the Coordination of Humanitarian Affairs), the Uppsala Conflict Database Program (a database that contains information on armed conflicts of the world since 1989) [5], and the Database on the Human Impact of Complex Emergencies (CE-DAT) [6]. Using the search terms "Congo and Mortality and Conflict" yields a total of 11 relevant articles. Three articles are commentaries on the war [7-9], 2 studies are from violence prior to the war [10,11], 1 study looks only at the Central and Western region [12], 1 examines displaced persons camps in a nearby Eastern province [13], 1 examines our setting of interest but is from 1999 [14], and 4 studies provide nationwide mortality estimates [15-18]. One of the 4 nationwide studies, a retrospective national survey, provides the most recent and comprehensive attempt to address the rates of mortality across the DRC [18,19]. You fail to find a more recent study specific to the North Kivu region.

The relevant article reports the 4^th ^mortality survey and 2^nd ^nationwide retrospective mortality survey conducted by International Rescue Committee. Conducted during April-July 2004, the survey inquired about deaths between January 2003 and April 2004 [18]. In general, retrospective mortality surveys select a sample of households; consenting households provide information regarding their demographic evolution over a given "recall" period of interest including all deaths, and their likely causes. The DRC study sampled 750 groups of households – termed "clusters" – representing 19,500 households. The study found a national Crude Mortality Rate (CMR) of 2.1 deaths per 1,000 per month (95% Confidence Interval [CI] 1.6–2.6), 40% higher than the remainder of Sub-Saharan Africa (1.5 per 1,000 per month) [1], and thus corresponding to 607,000 more deaths than one would expect in the population during the period of investigation [20]. Respondents reported fever and malaria, diarrhoea, respiratory infections and malnutrition as the immediate causes of more than 50% of deaths. Children under 5 accounted for 45% of all deaths. Mortality rates were higher in the Eastern conflict-affected provinces than the Western provinces (relative risk [RR] 1.3, 95% CI 1.2–1.5). Aware of the difficulties conducting research in unstable settings, you wonder about the accuracy of the data and how the results from this 2004 study apply to your current situation. The remainder of this article provides guidance to address this question.

## Introduction

Clinicians can now access well-established guides to facilitate optimal use of the medical literature [21]. In the realm of humanitarian emergencies, there have been, until recently, relatively few efforts to collect, report and appraise evidence. This dearth of evidence has resulted in confusion about the impact of war upon civilian populations. The poor quality evidence that does exist has, at times, been misused [22]. Thus, there is a pressing need for tools that clinicians and policy-makers can utilize in order to interpret the evidence effectively and apply the results in a judicious manner.

### The framework

In this paper we address the use of retrospective mortality surveys, a common form of measuring mortality in humanitarian emergencies [23]. Other methods can also be used, including routine mortality reporting and surveillance [24]. As with other articles in the Users' Guides series [25], we address the usefulness of an article through the following three questions.

#### 1) Are the results of the study valid?

This question considers whether the mortality estimates reported in the article accurately represent the magnitude of the problem. Another way to state this question is: Do the findings of the study represent an unbiased estimate of mortality in the given population over the period of time in which we are interested?

#### 2) What were the results?

To the extent that the results are valid, they will be worth applying to your public health scenario. Crucial to understanding results is the size and precision of the estimate. Reports will generally present best estimates of crude (all-age, all-cause, non-standardised), age-specific (children under 5 particularly), and cause-specific mortality. They may also present an absolute estimate of excess deaths.

#### 3) Will the results help you care for the population you are serving?

This question has several components. First, is the population studied similar to the population with whom you are working? Second, if the situation is similar, does the mortality study provide sufficient detail to assist in establishing your approach to health in the region? Finally, does the situation mandate humanitarian intervention beyond the medical care and public health strategies currently in place?

The text below summarizes our approach to evaluating and applying the results of articles assessing mortality in conflict-affected populations.

### 1. Users guide to an article about mortality in a complex emergency

#### Are the results of the study valid?

#### Primary guides

• Is the regional distribution of the sample studied sufficiently representative of the underlying population?

• Did the authors use random sampling to determine households or settings sampled?

• Do the investigators succeed in interviewing a large proportion of the chosen sample?

• Did the investigators institute specific strategies to ensure data accuracy?

• Did the study report revisiting households to confirm findings?

#### What are the results?

• How large is the mortality rate?

• How precise is the estimate of the mortality rates?

• What is the absolute death toll over the period of analysis?

#### Will the results help you care for the population you are serving?

• Can the results be applied to my setting?

• What are the specific causes of death?

• Can I corroborate these findings from local independent sources?

### 2. Using the guide

Returning to our opening public health scenario, how well did the study assessing nation-wide mortality in the DRC achieve the goal of representing the underlying population? The investigators tell us that they divided DR Congo into two strata along the 2001 line of military control: an east stratum of territory formerly held by rebel groups and a west stratum of territory formerly held by government forces. Within these strata, the investigators identified 511 health zones, and selected 4 through purposeful and 21 through random selection. Studies selected through purposeful sampling had been previously surveyed and provided historical comparisons. The investigators then selected 30 clusters in each health zone and visited 20 houses in each cluster in the West and 30 in the East, a total of 19 500 households and 119 378 people.

The investigators report using systematic random sampling in 186 clusters [24.8%]) that had detailed information on residents, and proximity random sampling in 564 clusters [75.2%] that did not.

Few households declined to participate in the survey: 22 (0.16%) in the east and only three (0.05%) in the west. The investigators tried to minimize non-response rates, and thus selection bias, by asking neighbours to assist in tracing the occupants of empty households. If they could not find occupants or if occupants refused to participate, or if no household member older than 14 years was at home, they skipped the household and visited the next nearest. Logistical, security, and time constraints prevented re-visiting empty households. They did not request independent confirmation of death.

### 3. Summary of key equations

$$\begin{array}{l} \text{Crude Mortality Rate }(\text{CMR})=\frac{\text{Number of deaths in the sample}}{\left(\text{Number living in sample}+\text{half deaths in sample}-\text{half livebirths in sample}\right)}\times \frac{10,000}{\text{Recall period}}\\ \text{Under-}5\;(<5)\text{ mortality rate }=\frac{\text{Number of deaths among those }<5\text{ years of age in the sample}}{\left(\text{Number living }<5\text{ years old}+\text{half deaths among those }<5\text{ years old}-\text{half livebirths}\right)}\times \frac{10,000}{\text{Recall period}} \end{array}$$

*Note*. The outcomes of interest in mortality studies are the rates and absolute numbers of deaths within a given population. Rates are usually expressed as a Crude Mortality Rate [CMR], most often as the number of deaths per 10,000 individuals in the population per day. The CMR provides the number of deaths per unit time within an at-risk community and considers the period of conflict and issues such as the number of births within a community, and even the number of people that come or go from a community (this calculation is not shown). Expressing the mortality outcome as a CMR provides much greater detail than simply reporting that, for example, '*100 adults were killed in recent weeks*' as the CMR provides inferences regarding the magnitude of death within a population within a specific time period. Clinicians and policy-makers can also apply the CMR to specific conditions, whether it is violent death, malnutrition, or malaria. Another rate often provided for interpretation is the Under 5 Mortality Rate (<5 MR) – that represents child and infant mortality, it is calculated similarly to CMR. Experts generally consider that a doubling of the CMR or <5 MR represents emergency status, although the definitions may vary across humanitarian agencies (See Table 1).

**Table 1:** Is this an emergency? [24]

**Agency**	**Assumed baseline**	**Emergency threshold**
Centres for Disease Control, Medecins Sans Frontiers Epicentre	Fixed at: CMR: 0.5 per 10,000 per day Under 5 MR: 1 per 10,000 per day	Emergency if: CMR: >1 per 10,000 per day Under 5 MR: >2 per 10,000 per day

UNHCR	Fixed at: CMR: 0.5 per 10,000 per day Under 5 MR: 1 per 10,000 per day	Definitions*: CMR: >1 per 10,000 per day 'very serious' CMR: >2 per 10,000 per day 'out of control' CMR: >5 per 10,000 per day 'major catastrophe' * Double each count for <5 MR

Sphere project	Context specific CMR (<5 MR) Sub-Saharan Africa: 0.44 (1.14) Latin America: 0.16 (0.19) South Asia: 0.25 (0.59) Eastern Europe, Former Soviet Union: 0.30 (0.20)	Emergency if: CMR (<5 MR) Sub-Saharan Africa: 0.9 (2.3) Latin America: 0.3 (0.4) South Asia: 0.5 (1.2) Eastern Europe, Former Soviet Union: 0.6 (0.4)

Definitions for emergency status thresholds

### Are the results of this study valid?

#### Is the regional distribution of the sample studied sufficiently representative of the underlying population?

In order for a mortality study to provide strong inferences about the impact of a conflict on civilian health, the study must represent the population at risk. Problems arise if a study over-samples a particular group; for instance over-sampling the male population in their twenties who are most likely to be involved in the fighting as either a member of a militia or as a victim relating to their potential for future militia roles will result in biased mortality rates [18,26].

The primary cause of death in conflict affected settings is often not directly related to violence, but rather to access and availability of health care, a lack of public health infrastructure and the emergence of malnutrition and serious diseases or exposure. Without access to basic needs like adequate nutrition, vaccination coverage or bed-nets, populations, particularly children, are at an increased risk for severe disease and subsequent death. In the study of the DRC, greater than 50% of all deaths were from non-violent causes; children under 5 proved the population suffering the largest percentage of deaths (45%) [18]. This finding is consistent with studies in conflict settings as diverse as Angola [27], Afghanistan [28], and Burma (Myanmar) [29]. If a study omits children in addressing population mortality, findings may exclude a large portion of deaths.

Determining whether the population studied is sufficiently representative of a conflict setting is challenging. An ideal study would use nation-wide census data that can provide region-specific demographic and mortality data. However, in many settings affected by conflict, populations are displaced, census data is out-of-date and Health Information Systems have been destroyed or lack staff [23,30]. The retrospective surveys that investigators conduct to remedy the problem may be not be representative of the at-risk populations. In many conflicts, the areas affected by conflict are regional and may prove difficult to access. If a survey targets an ethnic group, such as Karen and Karenni populations in Burma, that is a particular victim of violence, mortality estimates will be inflated [29]. If a study excludes populations directly affected by war, it will underestimate the mortality rates.

#### Did the authors report random sampling to determine households or settings sampled?

In humanitarian emergencies, complete household lists or even total numbers of households are often unavailable. The fall-back option in such cases is multi-stage cluster sampling. Clusters are groups of households sampled around a given number of starting points randomly spread through a primary sampling unit. Investigators divide the population into convenient sampling units (eg. districts, villages, camps). They then allocate the cluster starting points randomly among these units: if the probability proportional to size (PPS) approach is used, more populous units receive proportionately more clusters; alternatively, spatial approaches may be used, whereby units with the largest surface areas will receive the most clusters. Occasionally, cluster allocation occurs in several stages: the DRC survey first allocated clusters among different health zones, then distributed each zone's allotment of clusters among its villages. The total number of clusters will depend on the desired sample size; 30 clusters are generally believed to represent the minimum to permit adequate inferences that remain statistically sound [31]. Increasing the number of clusters is statistically preferable to increasing the number of households or individuals within each cluster as it provides greater interpretation of cluster-to-cluster variation, which is likely to be large in active conflict settings.

Because it is impossible to interview all households [32], surveys must sample the population in a way that avoids bias in the selection of individuals or households; random sampling is the best way to achieve this goal. If a list of all households with a unique identifier is available, the investigator can designate each household with a number and choose randomly from the household – an approach termed simple random sampling. If such a list is unavailable, but households are arranged in some geometric pattern and the investigators is aware of the number of households, the investigator can choose one household in a cluster at random, and then sample every *n*th contiguous household. In this second best option – termed systematic random sampling, the investigator chooses the interval between each household (the sampling interval) according to the desired sample size and by the total number of households [24]. A further option in emergencies consists of selecting a starting household at random (with a GPS unit, or a "spin-the-pen" random walk technique [33]), and sampling households around it via a rule of proximity (e.g. from door to nearest door).

The key question to ask about any survey sample is: did each household have the same chance of being selected? All of the above sampling designs, if performed diligently, will conform acceptably to this requirement. Occasionally, investigators interested in providing precise estimates of mortality rates in a specific group will intentionally violate the equal-probability rule by over-sampling that group. For example, the DRC investigators selected 20 households per cluster in the West, but 30 in the East of the country. This will still provide unbiased estimates if the analysis weights the results in proportion to the likelihood of sampling.

#### Did the investigators succeed in interviewing a large proportion of the chosen sample?

Failure to interview a large proportion of the target sample, either because households are not available or because they decline to participate, compromises the validity of the survey. Survey reports should present the proportion of non-responders (both those who were unavailable and those who refused), and reasons for unavailability. Decision rules are somewhat arbitrary, and the response rate is best interpreted according to whether non-responders may be systematically different from responders.

Furthermore, investigators can interview only households of which at least one surviving member remains, introducing the possibility of under-estimation of mortality due to entire households dying or disintegrating. This survival bias [34] is particularly likely when mortality is high, protracted, and focal.

### Did investigators institute specific strategies to ensure data accuracy?

Investigators should be asking detailed questions (eg. name, age sex, probably causes of deaths) about the specific members of a household to determine exact births and deaths, rather than simply summary counts. Inaccuracies in collecting reports of births and deaths may bias assessment of mortality rates. Interpretations of births or deaths may be inaccurate (eg. miscarriages) [35]; reports of death may be fabricated [35]. To overcome this difficulty, investigators may request birth or death certificates. Death certificates may vary in quality; some provide specific causes of death, others do not. In many settings lacking infrastructure, particularly among displaced populations, authorities will not issue death certificates, so investigators may ask health workers or elders to confirm deaths. Although asking for proof of death may be traumatic for family members, examples in which inaccuracy of death reporting led to misleading results suggest the need for this corroboration. For example, in a post-Gulf war study examining the impact of economic sanctions on mortality in children under 5 in Iraq, the study team reported an excess of 500,000 deaths [36]. When, a year later, the investigators repeated the study and matched a large proportion of the participants, they identified only 15% of the excess deaths [37], but not before national projections from this specious data was published [38]. Provision of death certificates may have avoided this extreme variability, which greatly weakens inferences from the data.

Interviewers, as well as respondents, may represent sources of misrepresentation. Investigators in a previous study in the DRC (2001) found that one of their interviewers had close ties to the rebel forces and under-reported deaths [24]. Current efforts to address such problems include: sending interviewers as teams, spot-checks of field forms, understanding the cultural definition of infant death, and comparing results among survey teams [24].

### Did the study report revisiting households to confirm findings?

An important strategy to reduce bias is to revisit a sample of households to ensure that the findings are replicable. As exemplified in the Iraq example of children under 5 [36,37], the reliability of a mortality estimate can vary widely. If one were to rely only on the first estimate from that study, as many have, one would incorrectly infer that 500,000 children had died [37]. More recently, the Iraq Living Conditions Survey, a large survey of 22,000 homes conducted in 2003/4, revisited a 10% of houses to inquire about specifically mortality in children under 5 and found about a greater number of births and deaths than initially reported [39]. Confirmation on resampling considerably strengthens results; failure to confirm, unless clearly explained, substantially weakens inferences.

Point 2 summarizes the validity assessment of our study in the DRC. The final assessment of validity is never a 'yes' or 'no' decision. Rather users can interpret the validity of a study as a continuum ranging from strong studies that are very likely to provide an accurate estimate of mortality to weak studies that are very likely to yield a biased estimate of effect. In this case, we judge that, despite limitations of corroboration and revisiting, we judge that overall, the study methods are moderate to high, and can provide some help to us in determining our medical response.

### What were the results?

#### How large is the mortality rate?

Most frequently, mortality studies estimate death rates over a specific period of time. Studies will take into account the number of new individuals born into the household as well as the number of individuals who have moved away. The study should then present the number of deaths as a mortality rate (See Textbox 1). The magnitude of the mortality rate can help us in 2 distinct ways:

(i) To quantify the extent to which excess mortality is occurring, by comparing the observed mortality rate with the best available estimate of the baseline mortality rate in the pre-war period, either by subtraction or by modelling a relative risk of dying in the post-versus pre-war period, if the recall period spans both.

(ii) To benchmark the severity of the crisis, by referring the study's mortality rate to internationally agreed-upon thresholds for defining states of emergency (Table 1). While several definitions for constituting an emergency exist (Table 1), baseline information will usually be unavailable [40]. The most widely accepted definition for Sub-Saharan Africa is the doubling of the regional baseline MR (~0.5 deaths per 10,000 per day), to approximately >1 per 10,000 per day [2,41,42].

#### How precise is the estimate of the mortality rates?

Surveys should provide confidence intervals [CIs], the likely range within which the true mortality rate actually lies. In practice, investigators usually use the 95% CI, the range that includes the true mortality rate 95% of the time. The narrower the CI, the closer the lower and upper boundaries of the CI are to the point estimate, the greater our confidence in the point estimate. In retrospective surveys employing cluster sampling, confidence intervals must be calculated taking into account the cluster sampling in order to get an accurate calculation of the sampling error.

The following 5 scenarios illustrate interpretation of confidence intervals:

1. Both the point estimate and the lower limit of the 95% CIs are clearly above the emergency thresholds of >1 per 10,000 per day (Eg. During the 1992 famine and civil war in Somalia, a large retrospective cluster survey (n = 5,200) of residents of Baidoa, Somalia, indicated a CMR of 16.8 per 10,000 per day with narrow CIs (14.6–19.1) [43]. Even if we believe only the lower CI boundary, the situation has reached UNHCR definition of 'out of control.'

2. The point estimate of the mortality rate lies above the emergency threshold. However, the 95% CI goes below our threshold for an emergency (Eg. Eastern DRC residents surveyed in 2002, CMR 1.2 (95% CI, 0.7–1.6) [44]. Our best estimate is that the CMR lies above our threshold, but it remains plausible that the true rate is appreciably below the threshold.

3. The point estimate is below the emergency threshold, but the upper 95% CI boundary crosses it (E.g. non-violent CMR among IDPs in Murnei Camp, Western Darfur, surveyed in May 2004, CMR 0.6 (95% CI, 0.4–1.2) [26]. Our best estimate is that the CMR lies below our threshold, but it remains plausible that the true rate is above the threshold.

4. Both the point estimate and the upper CI boundary are below the emergency threshold (Eg. Lugufu camp DRC IDPs in Tanzania, surveyed in 1998, CMR 0.4 (0.2–0.7) [45]. If the study is valid, one can be confident that the CMR is below the 1.0 threshold.

5. The CIs are so wide that the study provides little useful information (Eg. non-violent CMR among IDPs in Zalingei camp, Western Darfur, surveyed in April 2004, CMR 1.0 (95% CI, 0.3–3.1) [26]. The point estimate is on our threshold, but the truth may lie substantially below, or substantially above the threshold.

The study addressing the DRC found an overall CMR of 2.1 deaths per 1,000 per month (95% Confidence Interval [CI] 1.6–2.6). The CMR was increased in the Eastern regions 2.4 (2.2–2.7) and under 5 mortality in this region was 4.9 (4.4–5.4) per 1,000 per day. Only in regions reporting violence did the CMR per 10,000 per day (1.0 per 10,000 per day, 95% CI, 0.9–1.1) and under 5 MR (2.1 per 10,000 per day, 95% CI, 1.9–2.4) reach emergency definition status.

### How many people have actually died?

The magnitude of a mortality rate does not tell the entire story. What ultimately determines the actual death toll is how high mortality is, for how long and among how many people. In particular, we are interested in the total number of excess deaths. Investigators compute excess death tolls by multiplying the observed excess mortality rate (and its CI) by the length of the recall period, and by the estimated total population.

The DRC study authors assumed a baseline mortality rate for DRC of CMR 1.5 per 1000 per day, and subtracting this from the observed CMR of 2.1, obtained an excess MR of 0.6 per 1000 per day: this value, multiplied by the period (January 2003 to April 2004, or 16 months) and population (63.7 million) of which it is representative, yields an excess death toll of 607,000. Authors may also provide alternative figures based on different (more or less conservative) assumptions about the true baseline rate.

### Will the results help me care for my population?

#### Can the results be applied to my setting?

When interpreting mortality data we must question whether the population studied and the period of time that the survey took place might differ from our own situation. In order for findings to be useful, they should to be similar to the current situation or provide strong historical context that can assist in determining their relevance to the current context.

The impact of conflict upon a population can change dramatically depending on the progression of the conflict. If the conflict were to end abruptly, either through peace negotiations or one side being victorious, local health may improve or degrade almost immediately. For example, ethnic Tutsi mortality during the Rwandan genocide ended abruptly when Rwandan Patriotic Front soldiers won the civil war. Although mortality estimates would be dramatically different just a short period after the end of conflict, the studies remain useful for interpreting the historical context of patients' experiences.

#### What are the specific causes of death?

Most report causes of death, almost always as reported by next-of-kin of the deceased. The cause-specific-mortality rate expresses the proportion of death attributable to a specific cause. So, for example, in a large retrospective survey of IDPs in Angola in 2001/2002 the crude mortality rate in a region was 1.5 per 10,000 per day (95% CI, 1.3–1.8) and the proportion of death attributable to violence was 18%, then the cause-specific mortality rate for violence would be (1.5 per 10,000 per day × 0.18) 0.27 per 10,000 per day [27].

Cause specific mortality information is important for the development of an appropriate humanitarian response. A study assessing mortality in a large IDP (n = 175,000) population entering Murnei camp in Western Darfur in 2003–2004 found a CMR of 9.5 per 10,000 per day (95% CI, 6.4–14.0) and a proportionate ratio of 93% due to violence (cause specific MR attributable to violence, 8.9, 95% CI, 5.9–13.4) [26]. This finding provides inferences about the medical response and advocacy that may be required. In addition, this information can inform security concerns for both a regional response and international advocacy.

Although violence-specific deaths may be obvious, other causes of death are less so. The accuracy of cause-specific deaths is uncertain as many victims will have died without visiting a health provider. Further, verbal reports of cause of death are almost consistently inaccurate.

Determining whether the current context is similar to the context examined in a mortality study requires corroborating evidence, which leads us to our next question to determine if findings can and should be applied to the decision-making process required for intervention.

### Can you corroborate these findings from local independent sources?

In order to determine whether to apply the study findings to our field setting requires independent information from a variety of field-based sources. Local government and health authorities, other NGOs and international NGOs, clinicians working in ones' target setting, community leaders and elders, and community members of both genders may all provide useful corroborating information.

### Resolution of our public health scenario

The DRC mortality study addressed a large population of the DRC at a period in time where violence was subsiding in the West, but with sporadic periods of intense fighting in the East. Violence in the East remains problematic, despite the presence of UN troops in some regions. Many anticipated that recent elections in the DRC would result in progress towards peace, but violence, particularly in the North Kivu province remains high [46,47].

The study found that mortality related to violence in the region was a comparatively small contributor to overall mortality rates (1.5% of all deaths, cause-specific MR attributable to violence in Eastern region, 0.045 per 1000 per month, 95% CI, 0.028–0.072), but that settings with active violence had significantly elevated violence specific deaths compared to areas that did not report violence (CMR per 1000 per month is 1.7 times higher in violence-reporting communities (95% CI, 1.5–2.0, P = 0.001). Overall, most deaths reported in the study were from non-violent events including malnutrition (10.9% in the Eastern regions), fevers/malaria (27.4%), and diarrhoea (11.8%). In children under 5, deaths from meningitis (3.4%) and deaths in the neonatal period (9.4%) were reported higher in the east than in the west (1.5% and 5.5% respectively), whereas measles-related deaths appeared 1.63 times higher in the west. These findings suggest ongoing violence and a profoundly disrupted or debilitated health care delivery system in the Eastern regions, relative to other regions of the DRC.

By contacting local colleagues, conferring with community leaders and groups and observing patients presenting at our emergency clinic, we can confirm that current conditions remain similar to those described in the study. The DRC has received only 2.7% of the required USD 686,591,107 infrastructure budget it needs for 2007 so we can be reasonably sure that no major health infrastructures exist [48]. This information is useful to us now in reevaluating our approach to establishing our program and prioritizing populations, in particular children's health.

The information provided in this study provides a useful baseline with which to compare results from our own assessments, and will help better understand recent mortality at our clinic. These results, along with ongoing surveillance, help us in setting priorities for our health programs and in advocating with local and international actors for greater clinical services and increased security for the population at-risk.

## Competing interests

The authors declare that they have no competing interests.

## Authors' contributions

EJM, FC, JJO, MJS, FMB, CB, CC, CH, SS, RG, BAW, and GHG contributed in the concept, design, write-up, reviews, and have seen the final submitted manuscript. This work was initiated at a 1-day meeting to develop the Users Guide in Ottawa on March 8, 2007, supported by DFAIT.
